# Implementing at-birth, point-of-care HIV testing in Kenya: a qualitative study using the Consolidated Framework for Implementation Research

**DOI:** 10.1186/s43058-021-00188-9

**Published:** 2021-08-11

**Authors:** Catherine Wexler, Yvonne Kamau, Elizabeth Muchoki, Shadrack Babu, Nicodemus Maosa, May Maloba, Melinda Brown, Kathy Goggin, Natabhona Mabachi, Brad Gautney, Sarah Finocchario-Kessler

**Affiliations:** 1 Department of Family Medicine, University of Kansas Medical Center, Kansas City, KS USA; 2 Kenya Medical Research Institute, Nairobi, Kenya; 3 Global Health Innovations – Kenya, Nairobi, Kenya; 4 Health Services and Outcomes Research, Children’s Mercy Kansas City, Kansas City, MO USA; 5 School of Medicine, University of Missouri-Kansas City, Kansas City, MO USA; 6 Global Health Innovations – USA, Dallas, TX USA

**Keywords:** HIV, Early infant diagnosis, Point-of-care, Birth testing, Pediatric HIV, Kenya, Consolidated Framework for Implementation Science (CFIR), Qualitative

## Abstract

**Background:**

At-birth and point-of-care (POC) testing can expedite early infant diagnosis of HIV and improve infant outcomes. Guided by the Consolidated Framework for Implementation Research (CFIR), this study describes the implementation of an at-birth POC testing pilot from the perspective of implementing providers and identifies the factors that might support and hinder the scale up of these promising interventions.

**Methods:**

We conducted 28 focus group discussions (FGDs) with 48 providers across 4 study sites throughout the course of a pilot study assessing the feasibility and impact of at-birth POC testing. FGDs were audio-recorded, transcribed, and analyzed for a priori themes related to CFIR constructs. This qualitative study was nested within a larger study to pilot and evaluate at-birth and POC HIV testing.

**Results:**

Out of the 39 CFIR constructs, 30 were addressed in the FGDs. While all five domains were represented, major themes revolved around constructs related to intervention characteristics, inner setting, and outer setting. Regarding intervention characteristics, the advantages of at-birth POC (rapid turnaround time resulting in improved patient management and enhanced patient motivation) were significant enough to encourage provider uptake and enthusiasm. Challenges at the intervention level (machine breakdown, processing errors), inner settings (workload, limited leadership engagement, challenges with access to information), and outer setting (patient-level challenges, limited engagement with outer setting stakeholders) hindered implementation, frustrated providers, and resulted in missed opportunities for testing. Providers discussed how throughout the course of the study adaptations to implementation (improved channels of communication, modified implementation logistics) were made to overcome some of these challenges. To improve implementation, providers cited the need for enhanced training and for greater involvement among stakeholders outside of the implementing team (i.e., other clinicians, hospital administrators and implementing partners, county and national health officials). Despite provider enthusiasm for the intervention, providers felt that the lack of engagement from leadership within the hospital and in the outer setting would preclude sustained implementation outside of a research setting.

**Conclusion:**

Despite demonstrated feasibility and enthusiasm among implementing providers, the lack of outer setting support makes sustained implementation of at-birth POC testing unlikely at this time. The findings highlight the multi-dimensional aspect of implementation and the need to consider facilitators and barriers within each of the five CFIR domains.

**Trial registration:**

ClinicalTrials.gov, NCT03435887. Retrospectively registered on 19 February 2020

Contributions to the literature

- While at-birth and point-of-care testing can expedite infant HIV diagnosis and ART initiation, challenges to implementation in a real-world setting can cause delays and prevent scale-up.
- Using the Consolidated Framework for Implementation Research, our study is among the first to outline the process of implementation and systematically identify the multi-level facilitators and barriers to implementation and scale-up, from the perspective of implementing providers in Kenya.
- The data can be used to guide the implementation or scale-up of at-birth POC testing or similar interventions in low-resource settings.

## Background

Antiretroviral therapy (ART) by 12 weeks of age can reduce morbidity and mortality in infants living with HIV [1]. Early infant diagnosis (EID) of HIV services is critical to timely treatment. Conventional strategies for EID in Kenya require dried blood spot collection when the infant is 6 weeks and shipment to a central laboratory for processing. The mother is recalled to the hospital for result notification and, if positive, infant ART initiation. This process results in loss to follow-up between birth and testing, long turnaround times for sample processing, and loss to follow-up between testing and caregiver result notification [2–5]. Ultimately, only an estimated 63% of eligible infants/children are initiated on ART [6]. Many Kenyan infants diagnosed through EID services are not initiated until 17 weeks, reducing the benefits of very early treatment [7]. At-birth and point-of-care (POC) HIV testing for infants can streamline infant HIV testing and diagnosis. At-birth testing (using conventional polymerase chain reaction [PCR]) can reduce infant age at HIV diagnosis [8, 9] while POC testing can reduce turnaround times for results to < 1 day [9–14].

In 2016, Kenya updated their national prevention of mother to child transmission of HIV (PMTCT) and EID guidelines to recommend HIV testing for infants born to women living with HIV within 2 weeks of birth [15]; however, national implementation was delayed until piloting occurred. In 2018, the PMTCT/EID guidelines extended the pilot period for at-birth testing but recommended POC testing where already available [16]. As of the end of 2020, neither of these interventions had been widely implemented across Kenya.

## Methodology

From 2017–2018, our study team piloted and evaluated at-birth and POC HIV testing at four Kenyan hospitals [17]; however, we were unable to facilitate sustained implementation beyond the research period. Using the Consolidated Framework for Implementation Research (CFIR) [18], this study used thematic analysis to describe implementation from the perspective of implementing providers to identify factors that may impact scale-up outside of a research setting. CFIR combines constructs from a comprehensive review of implementation theories and constructs into a single framework containing constructs in 5 domains (intervention characteristics, outer setting, inner setting, characteristics of individuals, process) that comprehensively describes the factors influencing implementation.

### Description of parent study

Detailed descriptions of the parent study [17] and its results [10, 19] have been published. Briefly, pregnant women and/or mothers living with HIV who presented for PMTCT or EID services at study hospitals were eligible for inclusion in the parent study. All enrolled infants were eligible to receive POC testing using their hospital’s designated POC machine (AlereQ or GeneXpert) at two time points: < 4 weeks and 4–12 weeks of age. Samples for both AlereQ and GeneXpert were collected and loaded into cartridges and then processed. Processing for GeneXpert takes approximately 90 min, and Alere Q takes approximately 50 min. Conventional PCR samples were collected at the same time points and processed per standard procedures (dried blood spots collected and the hospital and then shipped to the central processing laboratory, processed, and then results sent back to the facility). Preliminary interviews with patients and providers conducted during implementation planning were used to inform implementation protocols [20, 21]. The objective of the parent study was to assess the feasibility and impact of implementing at-birth and POC testing for HIV-exposed infants in Kenya.

Prior to study implementation, clinical personnel from relevant hospital departments underwent a 2-day training. During implementation, site coordinators made periodic visits to implementing hospitals, supported research-specific tasks, assisted with patient follow-up, and contacted POC manufacturers when needed. Existing clinical staff conducted at-birth and POC testing, including counseling patients; sample collection, processing, and result notification at both time points; and treatment initiation, if applicable. Participant enrollment occurred from June 2017 to November 2018, with participant follow-up extending through March 2019. During the pilot period, providers were given a modest stipend for their role in study implementation (200 KSH [~US $2.00] per sample collected and processed).

### Qualitative procedures

We conducted 28 focus group discussions (FGDs) with providers at the 4 study hospitals (7 FGDs per hospital). Study hospitals were all government facilities and were in western (*n* = 2), coastal (*n* = 1), and central (*n* = 1) Kenya. Two were county-level hospitals, and two were sub-county–level hospitals. FGDs were conducted between December 2017 and April 2019. FGDs occurred approximately every 1–2 months; however, longer gaps occasionally occurred, around holidays or if providers were unavailable. Providers were purposely sampled to include those involved in implementing the pilot (identification of eligible patients, informed consent/counseling, sample collection/processing, patient management).

FGD size ranged from 4 to 10 participants, with most providers participating in multiple FGDs. FGDs occurred in a room in the hospital; were conducted primarily in English by trained study coordinators (NM, SB, EM) who had an existing relationship with providers, lasted approximately 1 h; and were audio-recorded. Written meeting minutes were kept during the FGDs. All participants signed a written informed consent prior to participation in their first FGD. Ethical human subject approval was obtained from the Kenya Medical Research Institute (SSC3390) and the University of Kansas Medical Center (STUDY00140399).

FGD guides were designed to use constructs from CFIR to assess the implementation of at-birth POC testing, including facilitators and barriers to implementation across the project lifespan, individual and stakeholder roles in project implementation, and suggestions to streamline and improve implementation. Guided by the CFIR guide question development tool [22], questions for each FGD were drawn from CFIR constructs and reflected the timing of implementation when that FGD was conducted. Questions were designed to query all CFIR constructs; however, if the result discussion shifted away from the intended construct, facilitators would often follow the new line of discussion, rather than transitioning back to the targeted construct; thus, not all constructs are represented in our results. Two authors (CW, MB) discussed and decided when (study launch, early implementation, mid-implementation, late implementation, throughout) questions should be asked to elicit responses, and some questions were asked in multiple focus groups to assess changes through implementation. FGD guides used for each meeting are included as supplementary material.

### Analysis

Audio recordings were transcribed verbatim. Four analysts (YK, SBK, NM, EM) used Dedoose [23] to independently code the transcripts for a priori themes, mirroring constructs from CFIR using the Dedoose analysis software. Analysts met periodically to develop and refine a codebook through iterative consensus building. Once coding was complete, two analysts developed memos for each code. Analysts met again to review each memo and discuss themes. An independent analyst (CW) reviewed all memos and codes to reach consensus on any disputed codes.

## Results

### Participant and FGD characteristics

In total, 48 providers participated across the 28 focus groups including 7 mentor mothers (MM, women living with HIV who have been through PMTCT/EID services and serve as peer health workers, provider counseling, and support provision of clinical services), 11 antenatal care (ANC)/PMTCT nurses, 2 HIV testing services (HTS) counselors, 5 maternal and child health (MCH) nurses, 4 maternity nurses, 6 clinical officers, 8 laboratory scientists, 4 nurses from the comprehensive care center (CCC, where specialized HIV care, including ART, is provided), and 1 data clerk. Table 1 describes the composition of FGDs across sites.

**Table 1:** Composition of the focus groups

Hospital ID (hospital level, location)	Average # participant (range)	Average # MM (range)	Average # nurse (range)	Average # lab scientists (range)	Average # clinical officer (range)	Average # others (range)
Hospital 1 (SCH, coast)	8.3 (6–10)	1.2 (0–2)	3.3 (3–4)	2.5 (2–3)	0.75 (0–2)	0.75 (0–1)
Hospital 2 (CH, western)	6.4 (6–7)	1.4 (1–2)	2.4 (1–4)	1.4 (1–2)	1 (0–2)	0.2 (0–1)
Hospital 3 (SCH, western)	5.2 (4–7)	0.8 (0–1)	2.8 (2–4)	1 (1)	0.6 (0–1)	0 (0)
Hospital 4 (CH, central)	6.4 (4–10)	1.6 (1–2)	3.6 (2–6)	0.7 (0–2)	0.1 (0–1)	0.4 (0–1)
Total	6.7 (4–10)	1.2 (0–2)	3.1 (1–6)	1.3 (0–3)	0.6 (0–2)	0.3 (0–1)

*MM* mentor mother, *CH* county hospital, *SCH* subcounty hospital

Of the 39 CFIR constructs and 5 domains, 30 constructs representing all five domains were discussed. Table 2 outlines the CFIR constructs and indicates the presence and salience of each construct within this study’s FGDs.

**Table 2:** Presence and salience of CFIR constructs with FGD themes

Construct	Salience^a^
Intervention characteristics	
Relative advantage	+++
Complexity	+++
Adaptability	+++
Evidence strength and quality	++
Intervention source	+
Cost	++
Design quality and packaging	–
Trialability	–
Inner setting	
Compatibility	+
Goals and feedback	+
Leadership engagement	+++
Structural characteristics	+
Implementation climate	–
Culture	+
Relative priority	+
Learning climate	++
Readiness for implementation	+
Access to knowledge and information	++
Networks and communication	+++
Organizational incentives and rewards	+
Available resources	+
Tension for change	–
Outer setting	
Patient needs and resources	++
Cosmopolitanism	+
External policy and incentives	++
Peer pressure	+
Characteristics of individuals	
Self-efficacy	+++
Knowledge and beliefs about the intervention	++
Individual stage of change	+
Individual identification with organization	–
Other personal attributes	–
Process	
Champions	+
Planning	+
Engaging	++
Executing	++
Reflecting and evaluating	+
Formally appointed implementation leaders	–
Opinion leaders	–
External change agents	–

^a^Salience was determined by the number of times each construct was coded: +, coded < 20 times; ++, coded 20–60 times; +++, coded > 60 times; –, not discussed within the FGD

The “Results” section of this paper is organized into main themes discovered in our results, with a discussion of applied CFIR constructs summarized for each section. The major themes discussed are facilitators to POC testing, barriers to POC testing, adaptations to implementation, suggestions for improved implementation, and suggestions to improve sustainability.

### Facilitators to POC testing

Facilitators to at-birth POC implementation fell within the CFIR constructs of relative advantage (intervention characteristics), patient needs and resources (outer setting), culture (inner setting), and relative priority (inner setting).

### Benefits of POC testing

The benefit of POC testing was a strong theme across FGDs and facilitated provider enthusiasm and support for POC testing. Providers felt that POC testing was a worthwhile intervention that improved patient care and management. Perceived advantages of at-birth POC included earlier testing and timeliness of results, leading to prompt and result-driven clinical management. Furthermore, early results increased patient motivation to stay engaged in care and reduced maternal anxiety:

It is of value to us in the management of our HIV patients and a motivator at the same time. I believe this is [easier] than waiting for the [conventional] PCR which was taking a bit of time. So, I believe the management is prompt. (H2_FGD5_Maternity In-charge)

Providers also described how POC alleviated caregiver anxiety since they got the results within a few hours, noting that this motivated caregiver’s adherence to ensure the baby continued being negative.

#### Hospital culture and relative priority

Given a hospital culture that cared for and prioritized their patients’ well-being, the recognized importance of EID programs and the perceived benefits of at-birth POC led providers to prioritize at-birth POC testing. Providers felt that POC testing both improved the services they were able to provide to patients and was an important contribution to national goals for HIV care and treatment. This facilitated ongoing cooperation and enthusiasm from providers even when challenges hindered implementation:

I think implementing POC is more important than other programs because, in maternity for example; when you deliver an HIV positive mother, and are able to link them into POC study, by the end of the day, we shall have helped to give the needed intervention for [elimination of mother to child transmission]. POC implementation therefore remains a priority. (H1_FGD3_CCCNurse)

### Barriers to POC testing

Providers discussed numerous barriers to conducting POC testing at the hospital level which mapped onto the following constructs: available resources (inner setting), compatibility (inner setting), complexity (intervention characteristics), patient needs and resources (outer setting), and structural characteristics (inner setting).

Resource constraints (available resources) were a frequently cited barrier to at-birth POC testing. The constraints most cited included shortage of trained staff, shortage of space, inconsistent electricity, and stockouts of cartridges. The shortage of staff to accommodate the increased workload posed a barrier to adding at-birth POC testing. In the post-natal ward, providers expressed concerns about inadequate space to provide confidentiality; while in MCH, concerns were raised about the lack of space for women to wait 1–2 h for their infant’s results to be available without disrupting the patient flow.

Power outages at sites with GeneXpert—which does not have a power backup—and at one site with AlereQ, whose broken power drum interrupted POC processing, complicated implementation, frustrated providers, and discouraged patients. Lastly, supply chain challenges resulted in a prolonged cartridge stockout, which disrupted the provision of at-birth POC.

Most hospitals assigned infant IDs at 6 weeks, which complicated the documentation of birth testing. While the study provided study-specific forms for documenting at-birth test results and clinical care, this testing strategy was not compatible with typical hospital documentation and needed to be created to utilize birth results for clinical care:

How do they document it in the [HIV exposed infant] register? And also in the management because the tools allow intervention from six weeks. But if it is now maybe [one] week, then it is bringing some confusion… (H2_FGD6_CCC in-charge)

Machine breakdowns occurred on several occasions and necessitated sending the machine for servicing and resulted in a temporary inability to conduct POC tests. Providers also explained their difficulty collecting adequate sample volumes from newborns. While these challenges occurred mainly at the beginning of the study and eased later in implementation, they were early complexities of implementation.

Patient-level challenges associated with at-birth and POC testing included the lack of fare to travel to the hospital, weakness, and needing time to recover after delivery. Patient disappointment was also noted when machine-related challenges prevented sample collection (stockouts) or processing (machine errors, power outages). Stigma, difficulties in disclosing to partners, and religious beliefs that dictated how soon the baby should be seen in public after birth also posed barriers to implementation: “Mothers who have not disclosed may have hard a time to justify their reasons for visiting the hospital.” (H1_FGD4_HTS counselor).

Providers from one high volume site described how the 1–2-h processing time was an inconvenience for patients, and how this was especially evident in situations when multiple patients needed testing or when results failed:

At times you would get two POC mothers so when you try to explain to the mothers that they will have to wait for 2 hours for the second POC client it becomes a challenge to convince them that they need to wait for the results. (H4_FGD2_ANC Nurse)

However, providers from other sites did not think the waiting time was a notable challenge:

And then most of them are so eager, they say they are not going home. They are waiting… (H3_FGD1_LabTech)

Hospital level also impacted patient follow-up, with the two larger, county-level hospitals more frequently citing challenges with patients who came for delivery being lost to follow-up by 6 weeks: “We are getting missed opportunities because we are a referral hospital. Some of them [patients] just come and after they have delivered they go back to their rural homes and then continue with PMTCT services.” (H2_FGD6_CCC In Charge). Providers from all hospitals spoke of challenges conducting the at-birth test when participants delivered elsewhere. Enhanced counseling throughout the ANC period was used to encourage patients to return to the hospital within 14 days for testing, regardless of delivery location; however, in some cases, this was not sufficient to overcome the patient-level challenges when delivery occurred elsewhere.

### Adaptations to implementation

Throughout implementation, sites needed to make changes to suit the context of their setup and improve intervention compatibility with existing hospital workflows. These adaptations can be most strongly mapped onto the CFIR construct adaptability (intervention characteristics); however, they are also related to the structural characteristics (inner setting) of each facility, impacted the complexity (intervention characteristics) and compatibility (intervention characteristics) of the intervention, and increased provider self-efficacy (characteristics of individuals) to implement the study.

While every facility had a similar structure, each facility was different in terms of proximity, location of machine, number of samples processed, workload, and space constraints. These affected implementation in terms of where the machine was located, who collected samples, who processed samples, and how various activities were coordinated. Machines were originally placed in the maternity department, but three of the four were moved to the hospitals’ on-site laboratory. While nurses were originally tasked with sample collection and processing, this responsibility often shifted to lab technicians once machines were moved to the laboratory. Even at the one hospital where the machine remained in the maternity department, lab personnel would travel to the maternity department to conduct the test to ease the already heavy workload of nurses, so they could focus on patient care.

Hospitals began assigning infants their ID number at birth—rather than 6 weeks—to simplify follow-up. Modifications to existing clinical registers allowed birth testing services to be documented with the rest of the infant’s clinical information. To protect patient confidentiality and reduce stigma, the timing of at-birth POC testing for newly delivered infants was adapted to protect patient confidentiality, “we do most of the testing early in the morning like it is the first thing, it is priority so by that time there no relatives” (H4_FGD4_PMTCT Nurse). To accommodate the wait time for the results and limited space for patients to wait, providers adapted their workflow to prioritize patients receiving a POC test, “We are also considerate of the time it takes for the POC result to be out, which is about 1.30 Hrs. So we can’t keep the patients in line waiting; they are directly ushered into the lab by the receptionist.” (H1_FGD4_HTS Counselor). Table 3 discusses the observed barriers and adaptations to address them.

**Table 3:** Observed barriers and adaptations to mitigate

Barrier	Adaptation/resolution
Space and human resource constraints	Re-locating POC machines based on hospital layout and availability of providers
Machine breakdown, errors	Enhanced training: this eased as providers gained more experience with the machine
Sample collection	Shifting sample collection to lab technicians. This eased as providers gained more experience
Assignment of infant IDs at 6 weeks	Modified hospital protocols to assign infant IDs at birth or first presentation
Patient difficulties traveling to hospital after delivery (fare, mother health, religious beliefs)	Unmodifiable^a^
Stigma, disclosure challenges	Conducting at-birth tests in the early morning, prior to visiting hours
Processing time for results	Prioritizing POC patients in queue to reduce their time at the hospital
Missed opportunities among home births	Enhanced counseling during the antenatal period

^a^Unmodifiable within the scope of the study

As the study progressed and these adaptations were made, providers felt that the intervention became more compatible with their workflow: “I see it as our normal routine. When mothers come, we retrieve their files and check if the results are in, or an infant is due for test etc. It’s just the routine process.” (H1_FGD3_MM). They felt that once they had gained experience using the machine, it was not complex. This learning curve was especially evident in the number of errors early in implementation: “You try, you get an error. And you think you did the right thing but still, you’re getting an error. You get another cartridge, you repeat the sample…I think nowadays we don’t get a lot of errors…” (H3_FGD7_MCH In-charge).

### Changing perceptions of POC

Throughout the course of the study, providers’ perceptions of POC testing changed. These changes can be strongly mapped onto the construct evidence strength and quality (intervention characteristics), knowledge and beliefs about the intervention (characteristics of individuals), individual stage of change (characteristics of individuals), and intervention source (intervention characteristics) and are more tangentially related to the constructs organizational incentives/rewards (inner setting) and peer pressure (outer setting).

At the beginning of the study, mistrust of POC results was evident with providers relying on conventional PCR results to guide clinical decision-making. By the end of the study, most providers believed that the benefits of at-birth POC testing outweighed the challenges. Consistently corresponding POC and conventional PCR results and their own observations of the impact it had on patients facilitated this transition and motivated continued implementation, “Some of the enrolled babies are now turning one year since the study inception, and the machine has continued to show consistent results. It’s a reliable machine.” (H1_FGD5_MM).

Several providers expressed pride that their hospital was among the first to be offering POC testing for infants. They saw this as an intervention that set their hospital’s quality of services apart from their peers, provided them with valued opportunities for career enhancement, and positioned their facility to serve as a learning center for other institutions: “It’s privilege and a great opportunity because we are stand as a hub for us to do point of care testing for all our babies at birth.” (H3_FGD4_Nurse in charge).

The intervention started as an external initiative, and providers were supported through small stipends for their effort. By the end of the study, many providers took ownership of the project and stated that they would be willing to continue implementing the intervention with reduced support from study staff: “So I think we’ve developed passion for birth testing and we are going to continue working even without the stipends.” (H3_FGD5_MCH Nurse).

Only a few stated that small stipends were needed for continued provider motivation. For these providers, an official hospital or national policy was needed to motivate continued implementation without financial incentivization:

It [cessation of stipends] will affect the study in a way… That is why if the study is to go full-fledged then it has to come from a circular so that everyone - so that it appears on the job description. So it is a responsibility that he is entitled to do, rather than that it is an extra duty we are told to do. (H2_FGD5_LabTech)

### Suggestions for improved implementation

Suggestions for improving implementation within the hospital centered around improving access to knowledge and information (inner setting), streamlining communication (inner setting: networks and communication) by making use of champions (process), and engaging (process) personnel outside of the immediate implementation team.

### Increase access to knowledge and information

Providers described how they felt that their access to knowledge and information was limited, both in terms of training and provided materials. Providers indicated the need for increased formal training. Providers noted that, while many people were trained, only a few actively participated in implementation:

The initial implementation was difficult because of the number of people trained versus the number of people doing the POC testing was not proportionate. Like almost 10 people were trained with only two people doing the testing so work was much (H4_FGD7_Maternity Nurse)

Providers described how trainings should engage departmental in charges and administration to ensure those selected for the training would participate. Providers appreciated the routine on-the-job training and site supervision provided by site coordinators; however, staff rotations and disinterest necessitated routine, formal training be conducted. Providers emphasized the need to train many clinicians in overlapping roles so that they would be able to support each other in times of heavy workload or if one was unavailable.

I think we need more trainings being that some of us have also left, the ones who were trained. So there is that gap and now the ones who were trained are few and some staff are also coming in. We need to work as a team. Because sometimes we can be busy, the other person can also be busy - the ones who were trained - so it’s hard if we need to do the test at that time. (H3_FGD4_LabTech)

Providers noted that manuals and printed materials helped resolve errors in the system; however, some indicated that study- or machine-specific materials were unavailable or inaccessible, “We need to have something like algorithm, a protocol to help us implement this system.” (H2_FGD5_LabTech). While some providers insisted that these materials were available, others at the same facility noted that they had not seen them, suggesting that provided materials did not always reach users:

And that is why I was telling [a colleague] I want that manual. [The colleague] has not given me that manual because it’s through that manual we know that this error means... (H3_FGD4_MCH Nurse)

Some providers noted that the printed training materials provided were not an effective way to spread information because “We are always busy running up and down. We are too exhausted to read them, by the time we are through with our shifts.” (H1_FGD5_LabTech). They suggested other methods of dissemination may include hosting continuing medical education seminars, on-the-job training, discussions, direct supervision, or email/soft copies—though these were not universally accepted as better methods.

### Streamline communication

The need for strong communication between clinicians in various roles and departments was a strong theme across sites and throughout the lifespan of the project. Given the interdepartmental nature of the intervention—involving personnel in the MCH, maternity, ANC, laboratory, and CCC—providers described how communication and collaboration across implementing departments helped reduce missed opportunities for testing, ensure smooth patient flow, and troubleshoot challenges. Providers described how early challenges with communication resulted in missed opportunities for testing, “you will find that MCH has drawn blood for testing and they did not call us to find out if there is a sample running so it just expires without it being tested.” (H4_FGD4_PMTCT Nurse). Furthermore, at the beginning, there was some friction between members of different departments, which hindered implementation, training and mentorship, and interdepartmental coordination. These conflicts often arose from perceptions that certain tasks were a certain person’s work: “Because the lab… they are technical staff. They don’t understand how [I] - a non-technical staff - can show them what to do… they don’t take it well.” (H3_FGD3_MM). As the study progressed and implementation logistics were adapted, this tension eased, with everyone comfortable with their role.

As the study went on, providers also developed strategies to streamline communication. While some hospitals chose to physically escort patients from one point to another and relay information to the receiving provider in person, respondents from other clinics observed that this was difficult if the distance was far or the workload was too high. Thus, these clinics suggested ways for real-time interdepartmental collaboration without physical movement such as having a dedicated phone or using discreet indicators on patient files. Timely communication necessitated identifying a “champion” to be a primary point of contact, which often fell upon a mentor mother.

Providers emphasized the need to ensure solutions to challenges went through the proper channels of approval so that they were accepted by the range of implementing personnel, “I think the best approach is the in charge i.e. the nurse in charge maternity and the departmental in charge…and…the in charge of HIV services in the hospital and see how best it can work out.” (H4_FGD5_MCH Nurse in charge).

### Suggestions to improve sustainability

Suggestions to improve the sustainability of the intervention centered around improving leadership engagement in the inner setting, increasing cosmopolitanism (outer setting) by better engaging outer setting stakeholders who would ultimately be responsible for funding (intervention characteristics: cost), and developing national-level policies to guide implementation (outer setting). Providing more formal goals and feedback at multiple levels and providing more opportunities for reflection and evaluation (process) were discussed as strategies to improve multi-level engagement and sustainability.

### Improved engagement of multilevel stakeholders

Despite their own enthusiasm, there was a general sense that the leadership within the hospital was supportive but hands-off, leaving implementation to the clinicians. While they may inquire about the progress of the study, they did not participate in decision-making, nor did they participate in routine meetings. Providers felt leadership engagement needed improvement to maintain operations outside of a research setting:

I would expect the people around us like in the administration like the MedSup [medical supervisor] to be part and parcel of the institutionalization. Because institutionalization require some internal memos that will require or mandate the teams responsible to make it a routine practice. (H2_FGD5_Maternity Nurse In Charge)

Similarly, providers felt that outer setting engagement was important for sustained implementation. The primary external connections were policymakers at the national and international levels, the county government, and implementing partners, who were tasked with the provision of HIV service at hospitals.

Providers discussed health officials’ critical role in sustaining POC implementation. County governments would be responsible for funding, provision of commodities, and hiring/training staff. Providers noted support from county health officials: “the county people are saying it is a good thing and it should be rolled out to all HIV exposed infants visiting the facility” (H4_FGD7_Maternity Nurse), but they expressed skepticism about the county’s ability to purchase the cartridges. Financing for commodities was predominant to conversations regarding sustained implementation after study wrap up, with the majority believing that “Cartridges are very expensive, and I’m not so sure if the county will manage to shoulder that.” (H1_FGD5_LabTech).

The need to include implementing partners more closely was mentioned by providers. These partners significantly contributed to hospital operations by hiring HIV staff and funding many HIV services. Without proper inclusion, providers believed they could hinder implementation. For example, staff hired by these partners may not feel that they could without risking their job:

You know, some of us are employed with the partner programs, and once they realize you’re working for…another program that is conflicting what they’re doing, then your job is also at stake. (H2_FGD7_LabTech)

Providers also discussed how supportive national guidelines would assist implementation in two ways. First, it would make implementation an official job duty and guarantee implementation, without financial incentivization. Second, it would guide clinical care in diverse situations.

If it is included in the guidelines then, even without staffing or without infrastructure, it will be a must, and every clinician, every lab person knows that POC is done…I mean, testing at birth is done to all babies, all HIV-exposed babies. (H2_FGD7_MM)

Overall, providers felt that a greater level of inclusion and involvement of partners external to the hospital was needed to support sustained implementation.

### Provision of feedback and evaluation

Providers noted that feedback occurred through the monthly meetings and FGD, which involved members of the implementation team. These meetings allowed providers to air grievances and provided an opportunity to discuss challenges and propose solutions, to express views, and to give feedback to the study team on whether they felt adequately informed and supported to conduct POC: “you have been able to call for the monthly meetings and update us on the study progress. That to me is a feedback….” (H1_FGD3_CCCNurse).

However, some providers felt that the feedback received during these meetings was inadequate to fully address the challenges and develop actionable plans for improvement:

The feedback we’ve been getting, I would say much needs to be done because some challenges are not fully explored and solved, though we get the feedback yes, but if we don’t explore more and solve some issues which are interfering with the performance then I think we will still remain at the same point. (H2_FGD4_Maternity Nurse-in-charge)

Providers noted the need for improved feedback to personnel outside of the implementing team, such as formal feedback on preliminary outcomes, which could be utilized at the hospital and county levels to support stakeholder buy-in:

Also it is very sad that we have that much job and there is no proper reporting…especially to the hospital. They don’t get the data they will not know what is going on and they will not know the importance of POC so that they can support (H4_FGD6_MM)

## Discussion

This study reported on qualitative, provider-perceived facilitators and challenges to at-birth POC implementation. Studies have indicated that at-birth POC testing can improve the timely return of infant EID results and ART initiation [11, 13, 14, 24]. Indeed, perceived improvements in patient care drove provider acceptance of and enthusiasm for at-birth POC testing in this study. Throughout the pilot, providers gained trust in the quality of the intervention. However, challenges (errors, stockouts, machine breakdown, patient-related challenges, communication challenges, staff turnover, or lack of interest)—especially in the early phases of implementation—caused frustration. Adaptions to implementation that occurred throughout the study period (modified logistics in terms of machine location, implementing personnel, workflow, and patient flow; improved mechanisms of interdepartmental communication; continuous OTJ training and site supervision) helped mitigate some of these challenges, but issues like power blackouts, machine breakdown, and high cost of cartridges will remain persistent barriers to sustainable implementation. These qualitative results support the quantitative evidence from our parent study [10, 19] that suggests that POC testing can expedite infant diagnosis, but challenges in POC implementation can result in missed opportunities for testing [10] and delayed ART initiation [19]. Providers felt that engagement beyond the implementing team was insufficient and discussed how more active engagement from stakeholders in the inner and outer settings was needed to continue implementation post-study.

Like many projects that fail to flourish beyond the pilot phase [25–28], providers cited numerous factors that were favorable to implementation but believed that sustainability and scale-up outside of a research setting was improbable. While factors related to the intervention (perceived advantages to patient care, alignment of intervention with national goals, ability to adapt aspects of implementation to their setting), characteristics of implementing individuals (their own self-efficacy to implement the intervention, their belief regarding the value of the intervention, and their own enthusiasm for the intervention), and inner setting (structural characteristics, strong communication between departments by the end of the study) were present to support implementation, other key factors were missing, namely, lack of leadership engagement at the hospital, county, and national levels and lack of clear external policies and guidelines for at-birth POC testing.

Our study team tried to comprehensively engage key personnel at different levels throughout the implementation process: from conducting pre-intervention formative interviews with stakeholders to assess support and guide protocol development (as previously described [20, 21]) to holding both sensitization (pre-study) and result dissemination (post-study) meetings at the county and local levels. To engage partners in the outer setting, the in-country PI shared study progress with administrators, county health officials, local implementing partners in the region and participated in the national technical working group on POC HIV testing. While providers at each hospital discussed their implementing “champions” who were critical for their role in coordinating testing activities on the ground, these champions often lacked the decisional power that would be needed to sustain the intervention. Thus, the need for champions at different levels to support various aspects of the implementation process was apparent. Ultimately, this lack of multi-level support and prioritization created a sense among providers that the high cost of cartridges would not be supported. As suggested by providers, we recommend the provision of formal quantitative and qualitative feedback at multiple levels (provider, hospital, county, and national) and at multiple points throughout the implementation process, which may help generate more upper-level ownership and support for the initiative.

Other POC pilot projects initiated in Kenya around the same time provided stakeholders with similar efficacy and impact feedback but experienced similar challenges with sustained implementation. For example, a joint EGPAF/CHAI project that supported 67 machines throughout Kenya from 2017 to 2019 [14, 29] continues to engage national and county stakeholders regarding sustained POC implementation. Despite technical assistance and support from PEPFAR Kenya, only 17 of the 67 machines had been used in the calendar year 2020 by July of 2020 [2]. The need to balance intervention impact and national priorities with limited funding in low-resource settings requires policymakers to make difficult decisions about which efforts to fund [30]. At this time, stakeholders at the county and national levels seem reluctant to sustain POC beyond a research/pilot setting. While POC testing was ultimately recommended in Kenya’s 2018 national guidelines, it was only recommended in settings where POC testing was already available, without the provision of additional funding or more specific algorithms/guidelines for implementation [16]. This presents an opportunity for the county and national stakeholders to engage implementing partners in supporting the 71+ under-utilized POC machines already available in Kenyan hospitals. The push for higher quality standards of care is a continuous process. Thus, based on the challenges identified in this study, we recommend frequent and continued engagement with key stakeholders, even after project completion—even after project completion—so that if priorities and funding opportunities become favorable to support implementation on a larger scale, those essential early engagements are already underway.

The challenge to engage the right people in the outer setting and within the inner setting throughout the implementation process came up in other ways throughout the FGDs. In planning implementation, our team started by engaging the county health director, who facilitated introductions with study site administration who then pointed us to the departmental heads who recommended 10–20 providers to be trained. However, FGDs revealed that despite these relatively large trainings, only a few providers actively participated, and many felt that the right people were not trained. Similarly, we provided study protocols, algorithms, and SOP to the heads of department; however, these may not have been accessible to those actively implementing the intervention. As we learned more about hospital contexts, we needed to continuously assess and change who should be engaged—and how this engagement should occur—at multiple levels. The CFIR model discusses how interventions have both core components and adaptable periphery components. Our study further supports the body of evidence that this adaptable periphery (logistics of implementation) is essential to making interventions relevant and feasible in various contexts.

CFIR provided a useful structure to provide a broad description of the implementation of at-birth POC testing. Our study was designed with the CFIR model in mind, with questions developed to explore each construct. Even so, not all constructs were explored in all FGDs, and some were not discussed at all. Other constructs were explored in detail during multiple FGDs, representing the constructs and issues most salient to providers. Our reporting on selected salient constructs demonstrates CFIR’s flexibility and versatility, yet limits cross-study comparisons, which is a noted limitation of CFIR [31]. Integration of CFIR from FGD design to outcome analysis is a strength of this study, as is the longitudinal nature of the study, allowing for a more representative picture of implementation characteristics throughout the pilot period, particularly when specific constructs may be more relevant or easily measured at various stages of implementation.

Limitations of this study include that providers simultaneously conducted both conventional PCR and POC tests, which is not representative of a real-world implementation, created additional work for providers, and thus may not be generalizable outside of a study setting. Second, the inclusion of only implementing clinical providers in FGDs gives a narrow picture of the implementation and likely swayed our results to over-represent the inner setting. Future studies should assess perspectives of non-implementing stakeholders, including patients, clinicians from peripheral departments (e.g., outpatient department), administrators, county health officials, national policymakers, and laboratory scientists at central laboratories where conventional PCR samples are sent.

## Conclusions and recommendations

At-birth POC testing for HIV has the potential to be a high-impact intervention that has proven feasible and acceptable at the inner setting and aligns with provider, hospital, and national priorities for HIV care. Even with inner setting support, such services cannot flourish beyond small pilot or research projects without adequate outer setting support. Our study was successful in implementing POC testing in hospitals and generating enthusiasm among implementing providers. Failing to facilitate pathways for sustainability and scale-up highlights the multi-dimensional aspect of implementation and the need to consider facilitators and barriers within each of the five domains of the CFIR model. Our recommendations for similar implementation projects include (1) maintaining flexibility in implementation procedures to allow the “adaptable periphery” to be adjusted to suit various contexts and (2) engaging multi-level key stakeholders from project conception through completion (and beyond, if necessary), including the provision routine, formal feedback on progress, successes, challenges, and efficacy.

## Data Availability

The datasets generated and/or analyzed during the current study are not publicly available due to the restrictions by the Institutional Review Board at the University of Kansas Medical Center but are available from the corresponding author on reasonable request.

## Abbreviations

ANC: Antenatal care
ART: Antiretroviral therapy
CCC: Comprehensive care center
CFIR: Consolidated Framework for Implementation Research
EID: Early infant diagnosis
FGD: Focus group discussion
FGDs: Focus group discussions
HIV: Human immunodeficiency virus
HTS: HIV testing services
KSH: Kenyan shillings
MCH: Maternal and child health
MM: Mentor mother
PCR: Polymerase chain reaction
PMTCT: Prevention of mother to child transmission
POC: Point-of-care
